# Studien zu neuen Immuntherapien: Herausforderungen aus Sicht der Ethik

**DOI:** 10.1007/s00103-020-03232-6

**Published:** 2020-10-16

**Authors:** Norbert W. Paul

**Affiliations:** Institut für Geschichte, Theorie und Ethik der Medizin, Universitätsmedizin Mainz, Am Pulverturm 13, 55131 Mainz, Deutschland

**Keywords:** Adaptive Studiendesigns, Umbrella-Studien, Basket-Studien, Ethik, Risiko-Nutzen-Erwägung, Adaptive study design, Umbrella studies, Basket studies, Ethics, Risk-benefit analysis

## Abstract

Neue Immuntherapien werden aufgrund der immer weiter reichenden molekularen Differenzierung von Erkrankungsmustern immer häufiger in sogenannten adaptiven, also fortlaufend an Ergebnisse angepassten Studiendesigns (Umbrella- oder Basket-Studien beziehungsweise Plattformstudien) klinisch erprobt. Der hier vorgelegte Beitrag diskutiert diese Studiendesigns jenseits der Feststellung von Regulierungsbedarf, um ausgehend von typischen Strukturmerkmalen ethische Probleme zu identifizieren und – wo möglich – Lösungsvorschläge zu machen. Neben dem Verhältnis von wissenschaftlichen und sozialen Werten in klinischen Studien werden insbesondere die wissenschaftliche Validität von Evidenz, Fragen des Einschlusses von Studienteilnehmern unter der Bedingung von relativer Unsicherheit, spezifische Herausforderungen für die ethische Bewertung adaptiver Studien sowie die ethischen und praktischen Herausforderungen im Bereich der Patientenaufklärung und -einwilligung in den Blick genommen.

## Abstract (en)

Novel immune therapies are more and more based on the molecular differentiation of disease patterns and related clinical studies are thus more often characterized by so-called adaptive study designs (umbrella or basket studies including platform studies), which are continuously adjusted based on novel results. This paper analyses new study designs beyond the often-postulated need for regulation in order to identify ethical problems based on typical structural features and to – whenever possible – suggest solutions. Additionally to the relationship between social and scientific values of a study as well as aspects of the scientific validity of new forms of evidence, the inclusion of study subjects under the condition of relative uncertainty, specific challenges in the process of ethical approval, as well as ethical and practical challenges in the process of informing patients and receiving informed consent will be addressed.

## Zur Ausgangslage

Die Prüfung und Zulassung neuer Wirkstoffe für den therapeutischen Einsatz hat sich in Deutschland von einer reinen Anzeigepflicht für neu auf den Markt gebrachte Medikamente bis in die 1960er-Jahre [1] hin zu einer behördlichen Zulassung nach dem Nachweis von Wirksamkeit, Unbedenklichkeit und idealerweise Überlegenheit neuer Präparate entwickelt. Randomisierte kontrollierte klinische Prüfungen (RCT) mit den typischen Phasen I–III basieren auf wohldefinierten Krankheitsbildern im Sinne von Krankheitsphänotypen. Nach klaren Einschlusskriterien werden Patienten in Studien aufgenommen und den jeweiligen Studienarmen und Kontrollgruppen zufällig (randomisiert) zugeteilt. Ziel solcher verblindeten Studien, d. h. Studien, in denen weder Studienteilnehmer noch die Durchführenden wissen, welcher Patient welcher Gruppe zugeteilt wurde, ist die Schaffung von Evidenz bezüglich der Sicherheit und Wirksamkeit eines neuen Präparats a) im Vergleich zu einer existierenden (Standard‑)Therapie mit oder ohne Placebogabe oder b) bei Fehlen einer vergleichbaren Therapie im Vergleich zu Placebo. Letzteres gilt nur, soweit eine Placebogabe bei einer erkrankten Kontrollgruppe aufgrund der Schwere der Erkrankung bei völliger Unwissenheit bezüglich der Überlegenheit der zu prüfenden Substanz (der ehrlichen Nullhypothese) ethisch rechtfertigbar ist.

Neuere Entwicklungen in der biomedizinischen Forschung haben zu einer molekularen Differenzierung von Krankheitsbildern und Patientengruppen sowie zu therapeutischen Ansätzen geführt, die nicht mehr vorrangig an einzelnen Krankheitsbildern orientiert sind [2]. Gerade im Bereich neuer Immuntherapien rücken molekulare und genetische Eigenschaften von Patienten oder Krankheitsprozessen – etwa bei Tumoren – als therapeutische Ansatzpunkte in den Fokus. Die zugrunde liegenden biologischen Strukturen und Prozesse finden sich typischerweise in unterschiedlichen Krankheitsphänotypen, im Falle der Onkologie etwa in unterschiedlichen Tumorentitäten. Damit sind traditionelle, auf eineindeutig definierte Krankheitsphänotypen bezogene Einschlusskriterien infrage gestellt. Zusätzlich wird durch die molekulare Präzisierung der therapeutischen Strategien eine Stratifizierung von Patientengruppen erreicht, sodass groß dimensionierte Studien mit vielen Teilnehmern unwahrscheinlicher werden und Studienarme kombiniert werden müssen, um die für aussagekräftige Ergebnisse erforderlichen Teilnehmerzahlen zu erzielen. Die ethischen Herausforderungen, die sich hieraus ergeben, werden im Folgenden auf adaptive Studiendesigns insgesamt bezogen [3]. Fünf ethisch relevante Charakteristika neuer Studiendesigns werden in den Blick genommen: 1. das Verhältnis von sozialen und wissenschaftlichen Werten in solchen Studien; 2. Fragen nach der Belastbarkeit und wissenschaftlichen Validität von Evidenz; 3. Fragen des ethisch balancierten Einschlusses von Studienteilnehmern; 4. Herausforderungen für die ethische Beurteilung von Studien im Rahmen ihrer Genehmigung; 5. spezifische Herausforderung in der Patienteninformation und informierten Einwilligung.

## Ethische Herausforderungen

Die Klärung des Verhältnisses von Zielen, Risiken und Nutzen in Studien zu neuen Immuntherapien kann als Lackmustest für eine klinisch erfolgreiche, ethisch rechtfertigbare und sozial akzeptable Implementierung neuer Studiendesigns verstanden werden. Wie ist dies gemeint? Es liegt in der Natur aktueller Immuntherapien, dass sie sich entweder mit unterschiedlichen molekularen Ansatzpunkten für ein und dieselbe Erkrankung oder aber umgekehrt mit der Wirkweise eines molekularen Ansatzpunktes in unterschiedlichen Krankheitsentitäten befassen. Um diesen Umstand abzubilden, sind in den letzten Jahren neue, von klassischen randomisierten, kontrollierten und doppelt verblindeten Studien (RCTs) stark abweichende Studiendesigns entstanden.

Die Untersuchung, auf welchen unterschiedlichen, gegebenenfalls auch kombinierten molekularen Pfaden eine einzelne (Tumor‑)Erkrankung angegangen werden kann, wird in sogenannten Umbrella-Studien abgebildet. Der Schirm (Umbrella), der sich über die verschiedenen in der Studie verfolgten Ansätze spannt, ist die gemeinsame Krankheitsentität. Die Untersuchung hingegen, wie ein einzelner molekularer Mechanismus immuntherapeutisch für unterschiedliche – wenn auch meist verwandte – Krankheitsentitäten wirksam gemacht werden kann, erfolgt häufig in sogenannten Basket-Studien. Der Korb (Basket) entsteht dabei bildhaft durch die therapeutische Strategie, in der unterschiedliche Patientengruppen etwa im Sinne molekularer Subpopulationen von Tumoren – z. B. Patienten mit Kolonkarzinom und Patienten mit Melanom – versammelt werden [4]. Der Vorteil dieses Studiendesigns liegt darin, dass unter einem Dach seltene oder sehr seltene Erkrankungen eingeschlossen werden können, für die Einzelstudien aufgrund der geringen statistischen Aussagekraft nicht sinnvoll sind. In Basket-Studien kommt es relativ häufig vor, dass unterschiedliche molekulare Patientengruppen verschieden auf therapeutische Interventionen ansprechen, woraufhin das Studiendesign zur Optimierung der Balance zwischen Risiken und Nutzen wie auch hinsichtlich der besseren Erreichbarkeit der definierten klinischen Endpunkte der Studie angepasst wird [4]. Diese klinischen Endpunkte, die letztlich das Kriterium für den Erfolg der Intervention bilden, werden in der Regel – auch in konventionellen onkologischen Studien – durch Surrogate, wie etwa die Zeit bis zum nächsten Rezidiv oder die Überlebenszeit, gebildet und nicht durch ein übergeordnetes Kriterium des Patientennutzens, wie etwa die Verbesserung der Fähigkeit zur sozialen Teilhabe im Sinne nichtstandardisierter Lebensqualität. Während man einerseits adaptive Designs aufgrund ihrer Reaktivität für vorteilhaft halten kann, entsteht durch sie andererseits eine Situation, in der die Beurteilung der Wirksamkeit von Interventionen, die Relevanz negativer Ergebnisse und die Aussagekraft der durch Adaption der Studie erzielten positiven Ergebnisse aus ethischer Sicht vorab kaum und im Verlauf der Studie schwer zu bewerten sind [5]. So stellen gerade adaptive Studiendesigns mit ihrer oft durch Anpassungen prolongierten Laufzeit und mit dem Ausscheiden nichterfolgreicher Ansätze sowie der Anpassung von Endpunkten eine Herausforderung dar, der, wie noch zu zeigen ist, wohl nur durch neue Verfahren der ethischen Bewertung und Begleitung begegnet werden kann [6].

### Soziale und wissenschaftliche Werte klinischer Studien

Sowohl in der wissenschaftlichen als auch in der ethischen Bewertung adaptiver Studiendesigns wird immer wieder auf das Kriterium der Equipoise verwiesen, wenn es darum geht, die Balance von sozialen und wissenschaftlichen Werten zu beschreiben. Um die ethischen Herausforderungen an dieser Stelle besser einordnen zu können, lohnt ein Blick sowohl auf die Konzepte der Equipoise als auch darauf, was mit den sozialen und wissenschaftlichen Werten einer Studie gemeint ist.

Im Jahr 1987 wurde durch eine Publikation von Benjamin Freedman das Konzept der klinischen Equipoise eingeführt. Dieses baut vor allem auf divergierenden klinischen Einschätzungen hinsichtlich der Überlegenheit einzelner Studienarme auf und veränderte damit die bisherige Sicht grundlegend [7]. Zuvor stand vor allem ein *theoretisches Konzept der Equipoise* im Fokus [8]. Dabei bedeutet theoretische Equipoise, dass diejenigen Kliniker und Wissenschaftler, die eine klinische Studie durchführen wollen, sich zu Beginn grundsätzlich im Unklaren darüber befinden sollen, welche der in den unterschiedlichen Studienarmen geprüften Behandlungsalternativen den anderen überlegen sein wird [9]. Nur so sei eine ethisch rechtfertigbare Randomisierung von Patienten in unterschiedliche Studienarme möglich; nur so könne die Randomisierung ohne wissentliche Benachteiligung einzelner Patientengruppen erfolgen [10]. Freedman ergänzte die theoretische Equipoise, die einen realitätsfernen Schleier der Unwissenheit bei klinischen und wissenschaftlichen Experten voraussetzt, durch das Kriterium der *klinischen Equipoise* [7]. Für diese ist es hinreichend, wenn eine ehrliche, professionelle Uneinigkeit unter klinischen und wissenschaftlichen Experten besteht, welche relativen medizinischen Vorteile von den jeweiligen unterschiedlichen Behandlungen der Studienarme zu erwarten sind [11]. Hier wird bereits deutlich, dass aus ethischen Erwägungen zur Vermeidung der Benachteiligung einzelner Patientengruppen eine Studie immer dann an einen Endpunkt kommt, wenn sich ein Behandlungspfad als überlegen erweist und damit das Kriterium der klinischen Equipoise nicht mehr zu halten ist.

Prinzipiell muss aus ethischer Sicht davon ausgegangen werden, dass eine klinische Studie immer nur dann gerechtfertigt ist, wenn Erkenntnisse gewonnen werden können, die mittelbar oder unmittelbar dazu beitragen, neue oder überlegene Therapien zu entwickeln oder bestehende Therapien hinsichtlich ihrer Wirksamkeit, Sicherheit und Verträglichkeit zu verbessern. Nebeneffekte können eine Verbesserung der Effektivität, etwa durch Verringerung von Interventionsintervallen und damit geringere Patientenbelastung, und der Effizienz im Sinne der Kostenreduktion sein. Diese beiden Effektgrößen *allein* reichen jedoch nicht aus, um die soziale Werthaltigkeit einer Studie zu belegen. Denn letztlich ist sowohl auf übergeordneter Ebene als auch innerhalb der konkreten Studie stets das umfassende Wohlergehen der Patienten [11] Dreh- und Angelpunkt der ethischen Rechtfertigbarkeit.

Idealerweise besteht zwischen dem sozialen und dem wissenschaftlichen Wert einer Studie eine ausgewogene Balance. Dies führt unmittelbar zu Überlegungen, wie Risiken und Nutzen einer Studie zu bewerten sind. Studienteilnehmer können einen direkten Nutzen, einen indirekten, auf eine Gruppe von Patienten bezogenen Nutzen (Gruppennutzen), einen Fremdnutzen (Nutzen für Dritte) oder keinen Nutzen von der Teilnahme an einer Studie haben. Letzteres ist in der Regel nur dann rechtfertigbar, wenn es keine Alternativen zu den in der Studie geprüften Verfahren gibt oder es sich um Studien an gesunden Probanden handelt. Ein negativer Nutzen, also eine Schädigung von Patienten durch studienspezifische Maßnahmen ist nur dann rechtfertigbar, wenn die Schwere und erwartete Häufigkeit der Schädigungen zumutbar gering sind (etwa Risiken studienbedingter Venenpunktionen). Die Zumutbarkeit – etwa durch körperliche, psychische und zeitliche Belastung oder durch Risiken von Eingriffen und Substanzen – ist in Abhängigkeit von Art und Schwere der Erkrankung und der Interventionstiefe zu bewerten.

Der Korridor für die Optimierung des Erkenntnisgewinns in einer Studie wird also auf der einen Seite durch die risikobezogene, ethische und soziale Rechtfertigbarkeit und auf der anderen Seite durch die Erreichbarkeit möglichst hoher Evidenzgrade definiert. Es ist exakt diese Stelle, an der neue adaptive Studiendesigns ansetzen. Ein Grund für die Kritik an klassischen Studien ist die potenzielle Gefahr, dass ein Teil der Studienteilnehmer in einem Arm der Studie suboptimal behandelt wird. Adaptive Studiendesigns versuchen dieses Problem dadurch zu lösen, dass vorläufige Studienergebnisse für Interimsanalysen genutzt werden, durch die Endpunkte der Studienprotokolls überprüft und angepasst werden [12]. Der Vorteil für Patienten besteht darin, dass sie zum Zeitpunkt des Einschlusses in die Studie bereits durch optimierte Verfahren behandelt werden. Ein Gerechtigkeitsproblem ergibt sich möglicherweise jedoch dadurch, dass diejenigen Studienteilnehmer, die zu Beginn der Studie behandelt wurden, gegenüber denjenigen Patienten, die erst spät oder sehr spät in die Studie eingeschlossen worden sind, einen Behandlungsnachteil haben, da bei Studienbeginn ja noch keine adaptiv optimierten Verfahren zur Verfügung standen. Hierauf wird noch einzugehen sein.

### Evidenz in adaptiven Studiendesigns

Ein immer wieder vorgebrachter Kritikpunkt gegenüber adaptiven Studiendesigns besteht hinsichtlich ihrer prinzipiellen Offenheit gegenüber systematischen Verzerrungen (Bias) durch fortlaufende Anpassung von Interventionen, Zwischenzielen und Endpunkten. Diesem wird in der Regel durch eine möglichst exakte Spezifizierung von Adaptionsoptionen vor Studienbeginn begegnet [13]. Die statistische Unter- oder Überschätzung von Ergebnissen ist aufgrund der oft komplexen Studiendesigns nicht immer einfach zu detektieren. Es ist sowohl aus klinisch-wissenschaftlicher als auch aus ethischer Sicht essenziell, einen Bias, der sich im Rahmen der Beobachtung, der Wiedergabe und Dokumentation von Ergebnissen, ihrer Analyse und letztlich einer möglicherweise selektiven Berichterstattung einschleichen kann, zuverlässig zu identifizieren [14].

Eine mögliche erste Fehlerquelle für die Integrität und Validität adaptiver Studien besteht darin, dass eine vollständige Verblindung, also das Unwissen darüber, welcher Patient welche Behandlung erfährt, nur schwer aufrechtzuerhalten ist. Dies gilt insbesondere dann, wenn die Studie aufgrund von Zwischenergebnissen angepasst wird. Es ist für Experten letztlich immer möglich, informiert zu raten, welchem Studienarm ein neuer Teilnehmer aktuell zugeordnet wird, zumal es sich ja teilweise um Studien mit sehr geringen Patientenzahlen handelt. Da es gerade im Bereich seltener Tumorerkrankungen zudem häufig keine wirksamen Alternativen der Behandlung gibt, mag ein zusätzlicher Bias dadurch entstehen, dass im klinischen Umfeld vorzugsweise diejenigen Patienten für eine Studienteilname in Betracht gezogen werden, von denen anzunehmen ist, dass sie von der Studienteilnahme profitieren.

Dies alles sorgt schließlich zusätzlich in klinischen Studien mit adaptiven Designs – insbesondere wenn es sich um (Immun‑)Therapien bei seltenen Tumorerkrankungen handelt – für eine eingeschränkte Replizierbarkeit und Reproduzierbarkeit, wodurch die Verallgemeinerbarkeit kompromittiert werden kann [15]. Letztlich verhält sich das Maß systematischer Verzerrungen umgekehrt proportional zum Maß der wissenschaftlichen Validität der geschaffenen Evidenz. Dies hat, wie leicht übersehen wird, nicht nur Auswirkungen auf die Qualität der Studienergebnisse. Evidenz kommt darüber hinaus a) eine rechtfertigende Funktion bei der Abwägung von Nutzen und Risiken und b) eine begründende Funktion bei der Einführung, Anwendung und Erstattung innovativer Verfahren in der Regelversorgung zu.

### Der ethisch balancierte Einschluss von Studienteilnehmern

Ein ethisch bedeutsamer Vorteil adaptiver Studiendesigns ist die Minimierung potenzieller Schädigungen von Studienteilnehmern durch eine Forschungsstrategie, die stets weniger Studienteilnehmer unter geringerer Unsicherheit Belastungen und Risiken der Studienteilnahme aussetzt. Durch fortlaufende Optimierung der Studien werden mehr Studienteilnehmer unter günstigeren Bedingungen, das bedeutet mit geringeren Behandlungsrisiken bei gesteigerter Erfolgsaussicht behandelt. Vor dem Hintergrund des im vorangegangenen Unterkapitel angesprochenen Bias beim Studieneinschluss von Patienten, die eine gute Aussicht auf einen Nutzen der Studienteilnahme haben, ist es zentral, Mechanismen zu schaffen, die eine faire und ethisch akzeptable Rekrutierungsstrategie sicherstellen. Dies wiederum stellt gerade für klinische Forscher, die in die Regelversorgung von Patienten involviert sind, eine normative Belastung dar.

Bei Fortschreiten einer Studie mit immer weiter optimierten Behandlungsstrategien kann es klinisch und ethisch zunehmend problematisch werden, die Anwendung einer überlegenen Therapie auf vorab selektierte Patientengruppen zu begrenzen, vor allem wenn Patienten, die spät in eine Studie eingeschlossen werden, von Ergebnissen, die mit früher eingeschlossenen Patienten erzielt wurden, profitieren. Man mag argumentieren, dass der Fortschritt, der sich sonst Schritt für Schritt in aufeinander aufbauenden Studien ergibt, hier lediglich in einer einzelnen Studie zusammengefasst werde. Es ist jedoch nicht trivial, wenn Teilnehmer unter unterschiedlichen Bedingungen, insbesondere hinsichtlich der Risiken und Nutzen, in eine Studienteilnahme einwilligen.

Dieses Gerechtigkeitsgefälle innerhalb einer Studie stellt derzeit gerade im Rahmen der klinischen Forschung zu Tumortherapien eine erhebliche ethische Herausforderung dar. Zum einen handelt es sich bei – insbesondere austherapierten oder therapierefraktären – Tumorpatienten um eine besonders vulnerable Patientengruppe mit einer in der Regel per se hohen Einwilligungsbereitschaft. Oft wird eine Studie als der rettende Strohhalm gesehen und nicht selten auch so präsentiert. Dem steht gegenüber, dass es gerade im Bereich der Immuntherapie durch adaptive Designs auch darum gehen kann, die am wenigsten effektiven Dosierungen und Kombinationen von Immuntherapeutika zu identifizieren und aus den aktiven Studienarmen zu eliminieren. Nicht alle Patienten dürften bei rückhaltloser Aufklärung in der Lage oder bereit sein, dies als vorwiegenden (Fremd‑)Nutzen ihrer Studienteilnahme in einem sehr frühen Stadium der Studie zu akzeptieren [15].

Damit wird das Eingangsversprechen adaptiver Studiendesigns, Teilnehmer hätten weniger Belastungen und Risiken unter den Vorzeichen geringerer Unsicherheit zu tragen, relativiert. Das komplexe Design von Studien im Bereich der Immuntherapie macht es daher immer häufiger erforderlich, dass in Basket- und/oder Umbrella-Studien eine größere Anzahl an Patienten länger in der Studie belassen werden muss, um im Rahmen von Interimsanalysen zu belastbaren Stichprobengrößen (Sample Sizes) zu gelangen [16]. Auch an dieser Stelle sind die Auswirkungen auf die Abwägung von Risiken und Nutzen der Studien evident.

### Herausforderungen für die ethische Beurteilung von Studien

In den vorangegangenen Abschnitten wurden bereits Kernelemente von adaptiven Studiendesigns im Bereich der Immuntherapie – mit einem vorrangigen Blick auf Tumorerkrankungen – benannt, die insgesamt relevant für die ethische Beurteilung dieser Studien im Rahmen der Genehmigung und der Erteilung von Ethikvoten sind. Allein die möglichen zukünftigen Änderungen im Rahmen einer einzelnen klinischen Prüfung sind derzeit nur schwer durch etablierte Verfahren der zuständigen Ethikkommissionen abzubilden. So kann es zur Öffnung neuer Studienarme, Schließung bestehender Studienarme, Änderung von Ein- und Ausschlusskriterien für Studienteilnehmer, Anpassung der Studienpopulation hinsichtlich molekularer Subtypen ihrer Erkrankung, Fallzahlen, Dosierungen von Prüfsubstanzen, Endpunkten und Kombination von Endpunkten, Änderungen von Abbruchkriterien und der Randomisierung (auch durch Veränderung von Studienarmen) zu Anpassungen kommen [4, 14], die weder durch ein einmaliges Votum einer Ethikkommission noch durch eine Abfolge von sogenannten Amendments (Änderungsanträgen) vollständig abgedeckt sein dürften. Durch Teilgenehmigungen bis zu definierten Interimsanalysen hätten adaptive Designs aus Sicht der Ethikkommissionen eher den Charakter einer Reihung von Teilstudien. Auch wäre eine Abfolge von Amendments problematisch, da insbesondere die Voraussetzungen für die Patientenrekrutierung und die informierte Zustimmung durch Änderungen im Risiko-Nutzen-Profil regelhaft in einem Umfang angepasst werden müssten, der über ein einfaches Amendment hinausgeht. Was zunächst als regulatorische Hürde erscheint, entfaltet überraschenderweise beim Blick in den zugrunde liegenden Gesetzestext eine erhebliche ethische Sprengkraft. Im Arzneimittelgesetz (AMG) heißt es hierzu unter §40 (1).

Die klinische Prüfung eines Arzneimittels darf bei Menschen nur durchgeführt werden, wenn und solange … 2. die vorhersehbaren Risiken und Nachteile gegenüber dem Nutzen für die Person, bei der sie durchgeführt werden soll (betroffene Person), und der voraussichtlichen Bedeutung des Arzneimittels für die Heilkunde ärztlich vertretbar sind.

Der entscheidende Punkt liegt hier in der Vorhersehbarkeit von Risiken und Nutzen, die durch komplexe adaptive Designs ex ante nicht gegeben ist, sondern im Verlauf der Studie immer wieder neu erfolgen müsste. Aus ethischer Sicht bedeutet dies, dass es einer Kombination aus gestuften, an Interimsanalysen orientierten Zulassungen von Studien und ihrer einzelnen Arme mit einer fortlaufenden ethischen Begleitung der Studie zur aktuellen Einschätzung von Nutzen und Risiken geplanter Anpassungen bedürfte. Letzteres erfordert zumindest eine prospektive Anpassung der Ressourcen der behördlich organisierten Ethikkommissionen in Deutschland. Dies gilt sowohl hinsichtlich der Menge der zu begutachtenden Prozesse wie auch in Bezug auf die Dynamik der klinischen Forschung. Daher könnte eine Anpassung des Instruments der DSMBs (Data and Safety Monitoring Boards) hin zu einem „Data, Safety and Ethics Monitoring Board“ ein erster Schritt in die Richtung einer berichtspflichtigen *wissenschaftlich-ethischen* Begleitung sein. Alternativ wird derzeit vor allem im Rahmen von Tagungen und Kongressen – etwa im Arbeitskreis medizinischer Ethik-Kommissionen – darauf verwiesen, adaptive Studiendesigns könnten auch im Sinne klinischer Entwicklungsprogramme verstanden werden. Damit würden sie gemäß §9 der GCP-Verordnung (Good Clinical Practice) zu behandeln sein. Für die Arbeit der Ethikkommissionen in der GCP-Verordnung §8 (3 und 4) lautet der entsprechende Passus:

(3) Betrifft der Antrag eine klinische Prüfung, die im Geltungsbereich des Arzneimittelgesetzes nur in einer einzigen Prüfstelle durchgeführt wird, verkürzt sich die in Absatz 2 genannte Frist auf höchstens 30 Tage. Ist diese klinische Prüfung eine klinische Prüfung der Phase I, die als Teil eines mehrere klinische Prüfungen umfassenden Entwicklungsprogramms auf einer von dieser Ethik-Kommission zustimmend bewerteten klinischen Prüfung desselben Entwicklungsprogramms aufbaut, verkürzt sich die Frist auf 14 Tage. Diese Fristverkürzungen gelten nicht bei klinischen Prüfungen der in Absatz 4 genannten Arzneimittel.

(4) Bei klinischen Prüfungen von somatischen Zelltherapeutika und Arzneimitteln, die gentechnisch veränderte Organismen enthalten, verlängert sich die in Absatz 2 genannte Frist auf 90 Tage; eine weitere Verlängerung der Frist auf insgesamt 180 Tage tritt ein, wenn die zuständige Ethik-Kommission zur Vorbereitung ihrer Bewertung Sachverständige beizieht oder Gutachten anfordert. Für die klinische Prüfung von Gentransfer-Arzneimitteln beträgt die Frist höchstens 180 Tage. Für die Prüfung xenogener Zelltherapeutika gibt es keine zeitliche Begrenzung für den Bewertungszeitraum.

Komplexe adaptive Studien im Bereich der (onkologischen) Immuntherapie, die auch immer häufiger Arzneimittel für neuartige Therapien (ATMPs) wie etwa Zell- und Gentherapeutika in den Blick nimmt, als Entwicklungsprogramme zu behandeln, hätte demnach den Vorteil, dass Anpassungen im Design nicht in einer Abfolge von Amendments abgebildet werden müssten, sondern als integraler Teil des Entwicklungsprogramms mit einer kurzen Frist von 14 Tagen genehmigt werden könnten, solange keine Zelltherapeutika oder genetisch veränderten Organismen eingesetzt werden, was jedoch zunehmend der Fall sein dürfte. Zudem liegt der Kern der eigentlichen ethischen Herausforderung an anderer Stelle [17]. Ein positives ethisches Votum kann nur so weit reichen, wie die Abwägung von Risiken und Nutzen für Studienteilnehmer, die auch vor dem Hintergrund der Beurteilung von sozialem und wissenschaftlichem Wert einer Studie zu erfolgen hat, sich auf ein wohldefiniertes und konkretes, das heißt auch vollumfänglich bekanntes Vorgehen stützt. Prospektive ethische Einschätzungen unter der Bedingung der Möglichkeit sind normativ nicht ausreichend belastbar, stehen sie doch regelmäßig unter dem Vorzeichen unsicherer Prognose oder von Unsicherheit per se. Dies führt unmittelbar zum letzten Punkt unserer ethischen Analyse, den spezifischen Herausforderung komplexer adaptiver Studiendesigns für die informierte Einwilligung der prospektiven Studienteilnehmer.

### Spezifische Herausforderung für die informierte Einwilligung

Es mag zunächst verwundern, wenn ein Element klinischer Studien, welches den Einstiegspunkt in die Forschungsaktivität bildet, am Schluss dieses Beitrags behandelt wird. Die Begründung ist, dass die Patientenaufklärung und damit die Grundlage für die informierte Einwilligung des Patienten im Prinzip ein Kondensat des Studienprotokolls mit all seinen angestrebten sozialen und wissenschaftlichen Werten, den Verfahren zur Schaffung von Validität und Evidenz, dem ethisch balancierten Einschluss von Studienteilnehmern im Rahmen von Randomisierungen [18] und schließlich der berufsrechtlich und ethischen Unbedenklichkeit ist. Damit sind alle vorab behandelten Aspekte Voraussetzungen für die Befassung mit der Frage, wie sich innovative adaptive Studiendesigns auf den Einschluss von Studienteilnehmern durch informierte Zustimmung auswirken, relevant. Das Thema soll hier pointiert, aber keinesfalls untergeordnet behandelt werden. Die Interessen der Patienten, die Risiken und Beeinträchtigungen, die sie im Rahmen einer Studie zu erwarten haben, der potenzielle direkte Nutzen, der Gruppennutzen oder auch nur der Fremdnutzen sowie das Gebot der vollständigen und rückhaltlosen Aufklärung sind zentral für die ethische Bonität von Studiendesigns [19].

Patienten, die in einem frühen Stadium der Studie einer Teilnahme zustimmen, sind nicht notwendigerweise in der Lage, bereits abzusehen, wie sich der Studienarm, in dem sie sich später befinden werden, entwickeln wird. Damit ist eine spezifische Einwilligung in eine wohldefinierte Intervention gar nicht möglich, sondern es wird eher – vergleichbar mit dem Vorgehen bei der Überlassung von Daten und Biomaterial für Biobanken – eine sehr weit gefasste informierte Einwilligung erfordert, die sich gegebenenfalls auch auf mögliche Anpassungen im Studienprotokoll erstreckt, die zum Zeitpunkt der Einwilligung noch gar nicht bekannt sind. Deshalb ist sicherzustellen, dass potenzielle Studienteilnehmer die Unterschiede zwischen der von ihnen geforderten, sehr breiten Einwilligung unter relativer Unsicherheit und der spezifischen Einwilligung, wie sie im Rahmen klassischer klinischer Studien mit statischen Ein- und Ausschlusskriterien und vorab definierten Interventionen gefragt sind, vollumfänglich verstehen.

Studienteilnehmer können unterschiedlichen Behandlungen, der Veränderung oder dem Austausch von Prüfsubstanzen unterworfen sein. Dies ist auch in dieser Dynamik vollständig und rückhaltlos darzustellen, damit potenzielle Studienteilnehmer ihre Einwilligung unter diesen prinzipiell offenen Bedingungen abwägen können. Gerade in Studien, in denen die am wenigsten effektive Therapie erst noch ausgeschlossen werden soll, muss vermittelt werden, dass eine statistische Wahrscheinlichkeit besteht – und wie hoch diese ist –, dass Teilnehmer zumindest zunächst nicht die individuell optimale Behandlung erhalten. Schließlich muss klar kommuniziert werden, dass diejenigen Studienteilnehmer, die erst später in die Studie aufgenommen werden, möglicherweise einen höheren Nutzen zu erwarten haben.

## Schlussbemerkungen

Gegenwärtig verweisen ethische Analysen biomedizinischer Innovation häufig auf Regulierungsbedarf. Auf diese Weise werden evaluative oft zu rasch zu normativen Ansätzen. Im Hinblick auf innovative Verfahren im Bereich klinischer Forschung und Entwicklung muss die normative Analyse stets im Spannungsfeld von Interessen und Evidenzen abwägen. Trotz der erkenntnistheoretischen Herausforderungen, die Konzepte von Evidenz mit sich bringen, sind ethische Ansätze, die vorrangig an der evidenzbasierten Lösung systematischer Probleme orientiert sind, oft besser operationalisierbar. Dies gilt umso mehr, als die Evidenzlage in der Regel nicht so volatil ist wie die Interessenlage. Ziel des hier vorgelegten Beitrags war es daher, Einblicke in ethisch relevante Strukturmerkmale adaptiv gestalteter, komplexer Studien zu gewinnen, um so hinzuweisen auf:

- Bedingungen der Realisierung der sozialen und wissenschaftlichen Werte klinischer Forschung unter Maßgabe neuer Studiendesigns,
- die ethischen Besonderheiten der wissenschaftlichen Validität von Erkenntnissen aus adaptiven Studiendesigns,
- die ethisch relevanten Auswirkungen der strukturellen Merkmale dieser Studiendesigns auf die Rolle der Studienteilnehmer,
- die Herausforderungen für eine ethische (und rechtliche) Bewertung adaptiver Studiendesigns,
- die Neuartigkeit der Bedingungen für eine informierte Einwilligung potenzieller Studienteilnehmer.

Dort, wo die ethischen Abwägungen entscheidend für die Art und Weise sind, ob und wie klinische Forschung und Entwicklung vorangetrieben werden kann, ist neben der Analyse auch Pragmatismus geboten [20]. Die theoriegesättigte Bewunderung des Problems bei gleichzeitiger Abwesenheit von Lösungsvorschlägen kann angesichts der Chancen, die neue Ansätze für komplexe, adaptive Studien im Bereich der Immuntherapie von Tumorerkrankungen haben, kein gerechtfertigtes Vorgehen sein. Daher soll an dieser Stelle ein Fazit für die Praxis den Abschluss bilden:

- I. Chancen und Risiken neuer – insbesondere adaptiver – Studiendesigns werfen ein neues Licht auf ethische Grundfragen, werfen jedoch keine grundsätzlich neuen ethischen Fragen auf.
- II. Neben einer schwierigen Balance zwischen sozialen und wissenschaftlichen Werten von adaptiven Studiendesigns fallen diese auch durch spezifische Strukturmerkmale auf, die systematische Verzerrungen (Bias) begünstigen. Es werden nur ein offensiver und offener Umgang sowie die Implementierung von Werkzeugen zur Detektion von Bias insbesondere bei der Patientenselektion und der Studienadaption helfen, Verzerrung zu verhindern oder zumindest transparent zu machen. Der Tatsache, dass die neuen Studiendesigns aufgrund der dynamischen Anpassungen von Zwischen- und Endpunkten nur schwer wiederholbar sind und damit die Verallgemeinerbarkeit der Ergebnisse leidet, kann möglicherweise durch Disziplin im Studiendesign, durch die Beschränkung auf essenzielle Anpassung, den Verzicht auf mögliche, aber verzichtbare Veränderungen sowie durch eine Schärfung und Standardisierung statistischer Werkzeuge begegnet werden.
- III. Ein wesentliches Problem besteht in den zu Studienbeginn nicht vollständig vorhersehbaren Anpassungen. Dies führt neben einer fortlaufend erforderlichen Neubewertung von Chancen und Risiken auch zu spezifischen Herausforderungen bei der Rekrutierung und Aufklärung von potenziellen Studienteilnehmern.
- IV. Die Charakteristika adaptiver Studiendesigns stellen derzeit eine Herausforderung im Rahmen der Erteilung von Ethikvoten dar. Zwischen einer restriktiven Position, in der die Zulässigkeit von Studien im Sinne des Arzneimittelgesetzes zur Fragmentierung adaptiver Designs in Einzelstudien oder aber zur Einreichung einer Folge oft komplexer und umfassender Amendments führt, und der technisch-pragmatischen Lösung, solche Studien als klinische Prüfungen im Rahmen eines Entwicklungsprogramms zu behandeln, müssen für komplexe adaptive Studien mit potenziell langer Laufzeit neue Werkzeuge entwickelt werden. Neben an Interimsanalysen geknüpften Teilgenehmigungen ist hier insbesondere an eine Ausweitung von Drug and Safety Monitoring Boards (DSMBs) zu „Drug, Safety, and Ethics Monitoring Boards“ (DSEMBs) zu denken.
- V. Die besondere Vulnerabilität bestimmter Patientengruppen – etwa Patienten mit austherapierten oder therapierefraktären Tumorleiden – erfordert einen besonderen Aufwand, um sicherzustellen, dass eine aufgeklärte Einwilligung unter einer realistischen Einschätzung von Chancen und Risiken der Studienteilnahme erfolgt. Insbesondere aufgrund des oft komplexen Studiendesigns werden hier in absehbarer Zeit neue Formen der Aufklärung und Einwilligung, etwa im Sinne einer an Zwischenzielen orientierten, gestuften Einwilligung zu entwickeln sein.
